# Does health literacy promote COVID-19 awareness? Evidence from Zhejiang, China

**DOI:** 10.3389/fpubh.2022.894050

**Published:** 2022-08-17

**Authors:** Chun Chen, Tingke Xu, Youli Chen, Yue Xu, Lizheng Ge, Dingming Yao, Xuehai Zhang

**Affiliations:** ^1^School of Public Health and Management, Wenzhou Medical University, Wenzhou, China; ^2^State Key Laboratory for Oncogenes and Related Genes, Key Laboratory of Gastroenterology and Hepatology, Division of Gastroenterology and Hepatology, Shanghai Institute of Digestive Disease, Renji Hospital, School of Medicine, Ministry of Health, Shanghai Jiao Tong University, Shanghai, China; ^3^Zhejiang Provincial Center for Disease Control and Prevention, Hangzhou, China

**Keywords:** health literacy, COVID-19 awareness, China Health Literacy Survey, Anderson's model, logistic regression

## Abstract

**Background:**

Health literacy (HL) is considered a crucial determinant of disease prevention and control. However, the role of HL in the Coronavirus Disease 2019 (COVID-19) pandemic has not been studied using provincial representative data among Chinese residents. This study aimed to assess the association between HL and COVID-19 awareness among Zhejiang residents based on the 2020 China Health Literacy Survey (CHLS).

**Methods:**

The study was conducted among 5,596 residents aged 15–69 in Zhejiang using multistage, stratified, and probability proportional to size sampling. COVID-19 awareness and HL were assessed using the “Chinese Citizen Health Literacy Questionnaire (2020)” in Zhejiang. The covariates were divided into predisposing factors, enabling factors, and need factors according to Anderson's model. Data were analyzed by the chi-square test and logistic regression.

**Results:**

The study showed that HL and COVID-19 awareness levels of residents were 24.84% and 8.06%, respectively, in Zhejiang in 2020. After adjusting for covariates, residents with adequate HL were more likely to have better COVID-19 awareness (odds ratio [OR] = 5.22, 95% CI = 4.13–6.59, *p* < 0.001). Three dimensions of HL (knowledge and attitudes, behavior and lifestyle, and health-related skills) were associated with COVID-19 awareness. Additionally, COVID-19 awareness was associated with age, occupation, family size, annual household income, and chronic conditions.

**Conclusion:**

COVID-19 awareness is significantly associated with HL, suggesting that promoting HL is an important component of health education, disease prevention, and health promotion in response to the COVID-19 pandemic and even possible public health emergencies in the future.

## Introduction

The first cases of the novel Coronavirus Disease 2019 (COVID-19) were reported in December 2019 (1). Since then, this infectious illness has spread worldwide, such as wildfire, affecting 448,313,293 people globally and claiming 6,011,482 lives as of 9 March 2022 (2). The COVID-19 pandemic has surfaced as a threat in several aspects of life with profound economic, social, and political impacts (3–5).

To prevent the transmission of COVID-19, individuals have been advised to stay at home, observe optimal social distancing, and practice preventive measures, such as washing their hands frequently and wearing a mask (6). The effectiveness of these preventive measures, however, depends largely on people's adherence to rules, which in turn is greatly affected by their COVID-19 awareness (7). A previous study reported that being informed about the source, transmission routes, and susceptible populations of COVID-19 could help to enhance people's willingness to practice preventive measures and consequently avoid transmission rates (8). Thus, COVID-19 awareness may prove to be essential in combating the COVID-19 pandemic.

Several factors seem to logically influence peoples' COVID-19 awareness, such as education and socioeconomic status (9), one such factor is health literacy (HL) that has, compared to other known factors, received limited attention until now (10). HL refers to the ability to access resources to enhance one's health-related knowledge and to ultimately use this knowledge for health-related “decision-making” (11). Extant literature has shown that HL contributes to disease prevention and control (12–14). As a result, promoting HL is now a public health goal in many countries, and interventions to improve HL are often prioritized (15–18).

On a positive note, the HL level of Chinese residents is constantly improving (19). Taking the example of Zhejiang Province, 33.08% of residents achieved adequate HL in 2020, which is a 3.59% increase from 2019 and a 24.63% increase from 2008. This may be one of the reasons why Zhejiang Province could calmly respond to the pandemic and achieve a significant outcome in the COVID-19 pandemic prevention and control.

However, the relationship between COVID-19 awareness and HL has not been empirically proven. Understanding this relationship could enhance society's awareness of the importance of HL and also promote the prevention and control of the pandemic for everyone. Hence, this study is aimed to examine the association between HL and COVID-19 among Zhejiang residents based on the 2020 China Health Literacy Survey (CHLS).

## Methods

### Study design

This was a cross-sectional study conducted among the residents of Zhejiang, based on the 2020 CHLS. The study participants were permanent residents of Zhejiang, aged 15–69 years, and who had continuously lived in the survey areas for more than 6 months. We excluded those who aged below 15 because this age group has usually not completed primary compulsory education yet. Residents above 69 were also excluded from the study because this age group was more likely to have impaired cognitive function (20).

All information regarding the research, with researchers' contact information and the voluntary and anonymous nature of participation, was given to the research participants before they began the survey. Potential participants were asked to indicate that they had read and comprehended the written consent/assent information and agreed to participate before being redirected to the research survey questionnaire. The study was reviewed and approved by the Research Ethics Committee of the Zhejiang Provincial Center for Disease Control and Prevention.

### Tools used

We adopted the “Chinese Citizen Health Literacy Questionnaire (2020),” which includes three parts (Personal Characteristics, HL, and COVID-19 Awareness) and was issued by the Chinese Center for Health Education. Among them, the questionnaire about personal characteristics and HL required the whole sample to answer. The questionnaire about COVID-19 awareness was proposed in the context of the epidemic, and the Chinese Center for Health Education required the Zhejiang Province to randomly select eight counties (cities or districts) to answer it.

The first part was designed to collect personal characteristics, such as gender, age, marital status, education level, and occupation; place of residence, family size, and annual household income; and chronic conditions and self-rated health (SRH).

In the second part, HL was assessed using the Chinese Health Literacy Scale. The Chinese Center for Health Education has developed this scale based on the “Health Literacy of Chinese Citizens: Basic Knowledge and Skills” using the Delphi methods (21). This 50-item scale consists of three dimensions: (a) knowledge and attitudes, (b) behavior and lifestyle, and (c) health-related skills (22). There are three types of questions on the scale: true or false (with 1 point given for each correct response), single answer (a multiple-choice question with only one correct answer, where 1 point is given for each correct response), and multiple answers (a multiple-choice question with more than one correct answer, where two points are given for each correct response) (23). For the multiple-answer questions, a correct response was defined as one that contained all of the correct answers and none of the incorrect ones (20). The overall Cronbach's alpha of the scale was 0.95, and the Spearman-Brown coefficient was 0.94 (24).

In the third part, COVID-19 awareness was assessed using 12 questions, as proposed in the context of the COVID-19 pandemic. All questions were divided into four single-choice questions and eight multiple-choice questions: single-choice questions were assigned a score of 0 or 1 and multiple-choice questions were assigned a score of 0 or 2 according to the scoring method of the Chinese Health Literacy Scale. The questionnaire was tested for reliability and validity, and the results showed that it has strong internal consistency (Cronbach's alpha = 0.86) and split-half reliability (Spearman-Brown coefficient = 0.93).

### Sampling methods

The minimum sample size per layer carried out by each county (city or district) is calculated as *N* = $\frac{{\mu_{\alpha }}^{2}\times p\times \left(1-p\right)}{\delta^{2}}$ × *deff*. Based on the HL level of 29.49% in Zhejiang Province in 2019, *p* = 0.2949, we set the allowable relative error of 15% and the allowable absolute error δ = 29.49% × 0.15 = 0.0442, μ_α_ = 1.96, *deff* = 1 and calculated the minimum sample size of each layer to be 413. Considering that the invalid questionnaire and rejection rate would not exceed 10%, we concluded that at least 640 residents per county (city or district) needed to be surveyed.

This survey was conducted in Zhejiang Province between July and October 2020. This study used stratified multi-stage probabilities proportional to population size (PPS) sampling frame. The sampling strategy followed the national guideline (25) and a description using similar strategies can be found in an earlier study (22), which was done in the following five stages: (1) 26 counties (cities and districts) were identified in Zhejiang Province, (2) four townships were selected from each county (city and district), (3) two segments (residential blocks) were selected within each of the selected townships, (4) 100 households were selected from each segment through a complete residential address list of all existing households, and (5) one participant was selected from each of the selected households using the Kish's grid (26). Through the sampling method described above, a total of 18,866 participants were interviewed face-to-face to complete the questionnaire about personal characteristics and HL. Additionally, Zhejiang Province was required by the Chinese Center for Health Education to randomly select the participants in Ningbo, Shaoxing, and Lishui to answer the questions about COVID-19 awareness. Therefore, a total of eight counties (cities and districts) in Ningbo, Shaoxing, and Lishui were included, and the final sample size was 5,596 in this study.

### Study measures

The independent variable, HL, was assessed using the Chinese Health Literacy Scale. The maximum total score of the scale is 66 points, with the maximum total scores of the three dimensions being 28 (knowledge and attitudes), 22 (behavior and lifestyle), and 16 (health-related skills) points. A total score of 53 (80% of 66) points or above was considered to indicate adequate HL. A score of 0–52 was considered to indicate limited HL. The HL level was defined as the proportion of participants who had adequate HL out of the total number of participants. The judgment criterion for adequate HL in each dimension was ≥80% of the total score for the dimension (22, 24).

The dependent variable, COVID-19 awareness, was assessed by four single-choice questions and eight multiple-choice questions (Table 1). The range of COVID-19 awareness was 0–20, which was divided into limited COVID-19 awareness (0–15) and adequate COVID-19 awareness (16–20) according to the judgment criterion for adequate HL. The COVID-19 awareness level was defined as the proportion of participants who had adequate COVID-19 awareness out of the total number of participants.

**Table 1:** Questions about COVID-19 awareness.

**Item**	**Correct rate (%)**
1.How much body temperature does it take to the hospital?	36.72%
2.How many days of isolation are required after close contact?	91.07%
3.Who is susceptible to COVID-19?	39.12%
4.What kind of mask should be preferred when shopping?	60.83%
5.Who are the source of the COVID-19?	26.91%
6.What are the transmission route of COVID-19?	17.89%
7.What protective measures should be taken by individuals?	27.77%
8.What are the correct ways to wash your hands?	70.91%
9.What are the correct ways to wear a disposable medical mask?	46.93%
10.If you need to eat together, what is the right approach?	92.26%
11.What protection should be done by individuals in low-risk areas?	72.09%
12.What are the responsibilities of citizens to prevent the pandemic?	73.36%

The covariates were defined according to Andersen's model and were as follows: predisposing factors included gender (men or women), age (15–35, 36–59, or 60–69), marital status (single/widow/divorced or married), educational level (less than junior high school, junior/senior high school, college, or above), and occupation (technical/professional, commercial/service, students, manual, or unemployed/others); enabling factors included the place of residence (urban areas or rural areas), family size (1–2, 3–5, or >5), and annual household income (0–49,999, 50,000–99,999, 100,000–149,999, or ≥150,000 Yuan); and need factors included chronic conditions (no or yes) and SRH (very good, good, fair, poor, or very poor).

### Statistics analysis

Data were analyzed using the SPSS version 26.0. The cutoff for significance was defined as a two-tailed *p*-value of <0.05. The personal characteristics, HL, and COVID-19 awareness of the sample were statistically described as the composition ratio and frequency distribution table. In order to evaluate the factors of COVID-19 awareness, the COVID-19 awareness scores were dichotomized into two categories: adequate and limited. The chi-squared (χ^2^) test was used to compare the COVID-19 awareness levels among different characteristic groups. A series of logistic regression models were fitted to examine whether COVID-19 awareness was associated with HL and the dimensions of HL. At each model, we reported the following descriptors and statistics: odds ratio (OR), *p*-value, and 95% CI; −2 log likelihood (−2LL); and Nagelkerke *R*^2^. The *R*^2^ mainly explained how much the model can explain the variation in COVID-19 awareness. The −2LL is a crucial indicator of model evaluation and the smaller the value, the better the goodness of fit.

## Results

### Basic characteristics

Table 2 presents the participants' basic characteristics. A total of 5,596 individuals completed the survey, of which 91.06% had limited COVID-19 awareness. The gender of the participants was almost the same, of which women accounted for 50.12%; 36–59 years old accounted for the most 55.33%; nearly half of the participants had a junior/senior high school education; workers and farmers accounted for 62.29%; more than half of the residents lived in rural areas; the most common family size was 3–5; the number of people with a household income (0–49,999) was the largest; the prevalence of chronic diseases was 26.23%; more than 60% of the residents rated their health was very good or good; and ≈24.84% of the participants had adequate HL.

**Table 2:** The COVID-19 awareness level by participant characteristics.

**Variables**		***N*** **(%)**	**COVID-19 Awareness**		**χ^2^**	***p*** **-value**
			**Limited**	**Adequate**		
	Total	5,596 (100.00)	5,145 (91.94)	451 (8.06)		
**Predisposing factors**						
Gender	Men	2,786 (49.79)	2,558 (91.82)	228 (8.18)	0.12	0.733
	Women	2,810 (50.21)	2,587 (92.06)	223 (7.94)		
Age	15–35	937 (16.74)	785 (83.78)	152 (16.22)	104.66	<**0.001**
	36–59	3,096 (55.33)	2,881 (93.06)	215 (6.94)		
	60–69	1,563 (27.93)	1,479 (94.63)	84 (5.37)		
Marital status	Single/Widow/Divorced	919 (16.42)	826 (89.88)	93 (10.12)	6.30	**0.012**
	Married	4,677 (83.58)	4,319 (92.35)	358 (7.65)		
Educational level	Less than junior high school	2,002 (35.78)	1,905 (95.15)	97 (4.85)	183.00	<**0.001**
	Junior/Senior high school	2,703 (48.30)	2,520 (93.23)	183 (6.77)		
	College or above	891 (15.92)	720 (80.81)	171 (19.19)		
Occupation	Technical/Professional	272 (4.86)	196 (72.06)	76 (27.94)	211.11	<**0.001**
	Commercial/Service	1,105 (19.75)	991 (89.68)	114 (10.32)		
	Students	174 (3.11)	142 (81.61)	32 (18.39)		
	Manual	3,486 (62.29)	3,297 (94.58)	189 (5.42)		
	Unemployed/Others	559 (9.99)	519 (92.84)	40 (7.16)		
**Enabling factors**						
Place of residence	Urban areas	2,465 (44.05)	2,240 (90.87)	225 (9.13)	6.79	**0.009**
	Rural areas	3,131 (55.95)	2,905 (92.78)	226 (7.22)		
Family size	1–2	1,724 (30.81)	1,603 (92.98)	121 (7.02)	5.17	0.075
	3–5	3,279 (58.60)	2,992 (91.25)	287 (8.75)		
	>5	593 (10.59)	550 (92.75)	43 (7.25)		
Annual household income	0–49,999	2,274 (40.64)	2,147 (94.42)	127 (5.58)	61.82	<**0.001**
	50,000–99,999	1,409 (25.18)	1,300 (92.26)	109 (7.74)		
	100,000–149,999	1,017 (18.17)	927 (91.15)	90 (8.85)		
	≥150,000	896 (16.01)	771 (86.05)	125 (13.95)		
**Need factors**						
Chronic conditions	No	4,128 (73.77)	3,742 (90.65)	386 (9.35)	35.42	<**0.001**
	Yes	1,468 (26.23)	1,403 (95.57)	65 (4.43)		
SRH	Very good	2,227 (39.80)	2,030 (91.15)	197 (8.85)	18.93	**0.001**
	Good	1,618 (28.91)	1,465 (90.54)	153 (9.46)		
	Fair	1,499 (26.79)	1,410 (94.06)	89 (5.94)		
	Poor	211 (3.77)	201 (95.26)	10 (4.74)		
	Very poor	41 (0.73)	39 (95.12)	2 (4.88)		
**Independent factor**						
Health literacy	Limited	4,206 (75.16)	4,046 (96.20)	160 (3.80)	413.78	<**0.001**
	Adequate	1,390 (24.84)	1,099 (79.06)	291 (20.94)		
Knowledge and attitudes	Limited	3,493 (62.42)	3,375 (96.62)	118 (3.38)	274.87	<**0.001**
	Adequate	2,103 (37.58)	1,770 (84.17)	333 (15.83)		
Behavior and lifestyle	Limited	4,015 (71.75)	3,850 (95.89)	165 (4.11)	299.20	<**0.001**
	Adequate	1,581 (28.25)	1,295 (81.91)	286 (18.09)		
Health-related skills	Limited	4,218 (75.38)	4,036 (95.69)	182 (4.31)	324.13	<**0.001**
	Adequate	1,378 (24.62)	1,109 (80.48)	269 (19.52)		

Boldface indicates statistical significance.

### COVID-19 awareness level among different characteristic groups

The chi-squared (χ^2^) test was performed (Table 2) to compare the COVID-19 awareness level among different characteristic groups. As for predisposing factors, statistically significant differences were found in the age composition (χ^2^ = 104.66, *p* < 0.001), marital status composition (χ^2^ = 6.30, *p* = 0.012), educational level composition (χ^2^ = 183.00, *p* < 0.001), and occupation composition (χ^2^ = 211.11, *p* < 0.001). As for predisposing factors, statistically significant differences were found in the place of residence composition (χ^2^ = 6.79, *p* = 0.009) and annual household income composition (χ^2^ = 61.82, *p* < 0.001). Regarding need factors, statistically significant differences were found in the chronic conditions composition (χ^2^ = 35.42, *p* < 0.001) and SRH composition (χ^2^ = 18.93, *p* = 0.001). Concerning the dependent variable, HL was closely correlated with COVID-19 awareness (χ^2^ = 413.78, *p* < 0.001).

Regarding predisposing factors, a lower COVID-19 awareness level was more likely to be found among participants aged 60–69 years; those who were married; those with low educational levels; and manual workers. As for enabling factors, a lower COVID-19 awareness level was more likely to be found among participants who lived in rural areas and those with an annual household income of 0–49,999 Yuan. Regarding need factors, a lower COVID-19 awareness level was more likely to be found among participants with poor SRH and those with chronic diseases. Concerning the dependent variable, among participants with adequate HL, 20.94% had adequate COVID-19 awareness, whereas only 3.80% of participants with limited HL had adequate COVID-19 awareness.

### Factors affecting COVID-19 awareness

A series of logistic regression models were performed (Table 3) to investigate the relationship between HL and COVID-19 awareness and determine the contributions of these variables. In model 1, which included the predisposing factors (gender, age, marital status, educational level, and occupation), age, educational level, and occupation were significantly associated with COVID-19 awareness. The predisposing factors accounted for a small proportion of the variation (Nagelkerke *R*^2^ = 0.085). In model 2, which added enabling factors (place of residence, family size, and annual household income) based on model 1, age, educational level, and occupation were still significantly associated with COVID-19 awareness. Additionally, place of residence and annual household income were also found to be significantly associated with COVID-19 awareness. The Nagelkerke *R*^2^ was changed only by 0.009. In model 3, which added need factors (chronic conditions and SRH) based on model 2, in addition to the factors associated with COVID-19 awareness in model 2, chronic conditions were found to be significantly associated with COVID-19 awareness. The need factors explained an additional 0.001 of the variation. HL was finally incorporated into model 4 and was found to be very significantly associated with COVID-19 awareness after adjusting for covariates and accounted for a further 0.078 of the variation in COVID-19 awareness, where individuals with adequate HL were more likely to have adequate COVID-19 awareness than individuals with limited HL (OR = 5.22, 95% CI = 4.13–6.59, *p* < 0.001). Moreover, age, occupation, family size, annual household income, and chronic conditions were also found to be significantly associated with COVID-19 awareness. It can be seen that among many factors, HL is the most important factor to explain the variation in COVID-19 awareness.

**Table 3:** Logistic regression: factors affecting COVID-19 awareness.

**Variables**	**Model 1**		**Model 2**		**Model 3**		**Model 4**	
	**OR**	**95% CI**	**OR**	**95% CI**	**OR**	**95% CI**	**OR**	**95% CI**
**Predisposing factors**								
Gender (ref. = Men)	0.87	0.71–1.07	0.88	0.71–1.07	0.86	0.70–1.06	0.84	0.68–1.04
Age (ref. = 15–35)								
36–59	0.56^***^	0.42–0.75	0.54^***^	0.41–0.72	0.58^***^	0.43–0.77	0.56^***^	**0.42**–**0.75**
60–69	0.55^**^	0.38–0.80	0.53^**^	0.35–0.78	0.61^*^	0.41–0.91	0.67	0.44–1.03
Marital status (ref. = Single/Widow/Divorced)	1.33	0.98–1.81	1.30	0.95–1.77	1.29	0.94–1.77	1.20	0.87–1.65
Educational level (ref. = Less than junior high school)								
Junior/senior high school	1.13	0.85–1.52	1.09	0.81–1.46	1.06	0.79–1.42	0.83	0.61–1.13
College or above	2.11^***^	1.42–3.15	1.95^**^	1.30–2.92	1.87^**^	1.24–2.80	0.92	0.60–1.42
Occupation (ref. = Technical/Professional)								
Commercial/Service	0.36^***^	0.25–0.52	0.35^***^	0.24–0.50	0.35^***^	0.24–0.50	0.40^***^	**0.28**–**0.58**
Students	0.68	0.39–1.20	0.69	0.39–1.23	0.67	0.38–1.20	0.62	0.34–1.13
Manual	0.30^***^	0.20–0.45	0.32^***^	0.22–0.49	0.32^***^	0.21–0.49	0.41^***^	**0.27**–**0.63**
Unemployed/Others	0.33^***^	0.21–0.52	0.31^***^	0.19–0.50	0.32^***^	0.20–0.51	0.36^***^	**0.22**–**0.59**
**Enabling factors**								
Place of residence (ref. = Urban areas)			0.77^*^	0.63–0.94	0.78^*^	0.64–0.96	0.84	0.68–1.04
Family size (ref. = 1–2)								
3–5			0.89	0.69–1.14	0.87	0.68–1.12	0.80	0.62–1.04
>5			0.70	0.48–1.04	0.70	0.47–1.04	0.65^*^	**0.44**–**0.98**
Annual household income (ref. = 0–49,999)								
50,000–99,999			1.37^*^	1.04–1.80	1.36^*^	1.03–1.79	1.31	0.98–1.73
100,000–149,999			1.26	0.93–1.70	1.25	0.92–1.68	1.16	0.85–1.58
≥15,0000			1.61^**^	1.19–2.17	1.58^**^	1.16–2.13	1.42^*^	**1.04**–**1.95**
**Need factors**								
Chronic conditions (ref. = No)					0.65^**^	0.48–0.88	0.68^*^	**0.50**–**0.92**
SRH (ref. = Very good)								
Good					1.02	0.81–1.29	0.99	0.78–1.26
Fair					0.85	0.65–1.12	0.87	0.65–1.15
Poor					1.04	0.53–2.06	1.05	0.53–2.11
Very poor					0.99	0.23–4.27	1.25	0.28–5.57
**Independent factor**								
Health literacy (ref. = Limited)							5.22^***^	**4.13**–**6.59**
−2 LL	2927.67		2906.69		2895.03		2697.54	
Nagelkerke *R*^2^	0.085		0.094		0.098		0.176	

Boldface indicates statistical significance

* p < 0.05;

** p < 0.01;

*** p < 0.001.

After using logistic regression to control the covariates, the relationship between the three dimensions of HL and COVID-19 awareness was further analyzed. The results showed that knowledge and attitudes, behavior and lifestyle, and health-related skills were significantly associated with COVID-19 awareness (Table 4).

**Table 4:** Health literacy dimensions associated with COVID-19 awareness.

**HL Dimensions**	**Adjusted Models**		
	**OR**	**95% CI**	***p*** **-value**
Knowledge and attitudes	4.11	3.22–5.25	<**0.001**
Behavior and lifestyle	3.73	2.99–4.64	<**0.001**
Health-related skills	4.03	3.23–5.02	<**0.001**

Boldface indicates statistical significance.

## Discussion

The present work is, to the best of our knowledge, one of the first to examine the relationship between HL and COVID-19 awareness. Our study suggested that the HL played a critical role in explaining COVID-19 awareness in China.

The accuracy of questions on COVID-19 awareness differed greatly in this study, ranging from 17.89% (“What are the transmission routes of COVID-19?”) to 92.26% (“If you need to eat together, what is the right approach?”), which was considered to be significantly lower than that of other countries, such as Jordan and Iraq (27), Pakistan (28), Malaysia (29), and Bangladesh (30). However, because the types and difficulties of the questionnaire used in different countries were different, it is inappropriate to directly compare the accuracy rate.

Furthermore, the COVID-19 awareness level among individuals with different demographic characteristics was also significantly different, that is, it was lower among participants aged 60–69 years old, those with low educational levels, and manual workers, which was consistent with the results of other research studies (27, 31, 32). This could help the government better target people with lower COVID-19 awareness levels through the identification of demographic characteristics. Moreover, they could carry out timely health education to enhance the public COVID-19 awareness levels to strengthen its awareness regarding prevention and control and better respond to the impact of the COVID-19 pandemic.

Although age, occupation, family size, annual household income, and HL are found to affect COVID-19 awareness in model 4, HL explains the largest proportion of variation. Moreover, the three dimensions of HL were closely related to COVID-19 awareness. This conclusion supported the findings of previous studies in which HL played an important role in the prevention and control of many infectious diseases, such as vaccine-preventable diseases (33–36), sexually transmitted diseases (37), viral hepatitis infections (38), and tuberculosis (39).

Notably, a higher COVID-19 awareness level was significantly associated with adequate HL in this study. The finding clearly indicated the importance of improving COVID-19 awareness *via* health education, which could help individuals better understand its source, transmission routes, and susceptible populations. This would encourage people to pay more heed to preventive measures, such as wearing masks and washing hands frequently, and believe that we will eventually defeat the COVID-19 pandemic (28, 30, 40). Therefore, the results of this study not only provided new evidence for the importance of HL but also indicated the focus for subsequent responses to major public health emergencies *via* targeted health education and improvement of HL in the public.

Finally, if the public strives to improve HL unanimously, it is possible to enhance residents' COVID-19 awareness and control the pandemic's spread. Moreover, a public health emergency, such as COVID-19, may not be the last public health emergency to occur. Even though specific response measures would be different, improving the HL of residents could be an effective way to deal with future public health emergencies.

## Limitations

This study was fraught with some limitations. First, because a cross-sectional research design was adopted in this study, causal relationships between HL and COVID-19 awareness could not be examined. Future studies should be conducted to examine these relationships in detail. Second, potential correlates, such as the HL of health behaviors and health service quality, were not measured in this study. Further research is needed to examine the process of how HL influences COVID-19 awareness. Third, there is no globally accepted questionnaire scale for the measurement of COVID-19 awareness and, thus, the results cannot be compared. There is a need for more standardized questionnaires to be designed.

## Conclusion

This study showed that COVID-19 awareness is associated with HL even after adjustments for personal characteristics are made. Higher COVID-19 awareness level is more prevalent among Chinese residents with adequate HL. Furthermore, age, occupation, family size, annual household income, and chronic conditions are associated with COVID-19 awareness. HL not only helps to control the spread of the COVID-19 pandemic but can also be an important channel for the prevention and control of future diseases. Therefore, the promotion of HL should be an important component of disease prevention, health education, and health promotion.

## Data availability statement

The raw data supporting the conclusions of this article will be made available by the authors, without undue reservation.

## Ethics statement

This study was approved by the Research Ethics Committee of the Zhejiang Provincial Center for Disease Control and Prevention. All study procedures were conducted in accordance with ethical standards. Written informed consent was obtained from each participant or a legally designated representative.

## Author contributions

CC and TX are responsible for conceptualization, data collection, methodology, resources, and software. YC is responsible for conceptualization, data collection, and methodology. YX is responsible for methodology and software. LG is responsible for data collection and investigation. XZ and DY are responsible for conceptualization, funding acquisition, resources, supervision, reviewing, and editing the manuscript. All authors contributed to the critical revision of the manuscript for important intellectual content, review, and approval of the final manuscript.

## Funding

This project was sponsored by Zhejiang Provincial Natural Science Foundation (LQ16G030011), the Key Social Science Project for University Teachers of Zhejiang, China (2014QN005), and the Key Statistical Research Project of Zhejiang in 2021 (21TJZZ08).

## Conflict of interest

The authors declare that the research was conducted in the absence of any commercial or financial relationships that could be construed as a potential conflict of interest.

## Publisher's note

All claims expressed in this article are solely those of the authors and do not necessarily represent those of their affiliated organizations, or those of the publisher, the editors and the reviewers. Any product that may be evaluated in this article, or claim that may be made by its manufacturer, is not guaranteed or endorsed by the publisher.
